# Comment on Fogliazza et al. Approaches to Pediatric Chest Pain: A Narrative Review. *J. Clin. Med*. 2024, *13*, 6659

**DOI:** 10.3390/jcm14030742

**Published:** 2025-01-24

**Authors:** Carlos Cotrim, Nuno Cotrim

**Affiliations:** 1 Heart Center do Hospital da Cruz Vermelha, 1549-008 Lisboa, Portugal; 2 Cardiology Department do Hospital de Santarém, 2005-177 Santarém, Portugal; nuno_cotrim1@hotmail.com

We read with interest the excellent review manuscript of Frederica Fogliazza et al. [1]. This narrative review highlights that chest pain in children and adolescents is a frequent reason for emergency department visits and referrals to pediatric cardiologists, often driven by parental concerns regarding potential cardiac issues. However, most cases of pediatric chest pain are benign and non-cardiac in origin. The review discusses the causes, evaluation, and management of pediatric chest pain, emphasizing the value of a detailed clinical history and physical examination in distinguishing between benign and serious conditions. It also examines the role of diagnostic tests such as electrocardiograms, chest radiography, and echocardiography, emphasizing the need to balance avoiding unnecessary testing with ruling out life-threatening cardiac conditions. Although cardiac causes are rare, the variability in diagnostic practices underscores the importance of standardized evaluation algorithms. Such algorithms could streamline care, reduce unnecessary resource use, and alleviate patient and family anxiety. The authors give significant attention to SCAMPs [2,3,4] (Standardized Clinical Assessment and Management Plans), particularly noting the limited value of ECG exercise testing. They also highlight the necessity for future studies to evaluate the effectiveness of these algorithms in improving clinical outcomes and resource efficiency. Overall, the findings emphasize the importance of a careful, evidence-based approach to managing pediatric chest pain.

However, our primary concern is the lack of reference to exercise-induced intraventricular gradients, which are easily detectable through exercise stress echocardiography and associated with chest pain and other symptoms [5,6,7,8,9,10], even in children. These gradients should have been acknowledged as an area requiring further study.

As an example, we included an echocardiogram image of a 14-year-old female handball player recently evaluated by our group, who experienced angina (Figure 1) before starting bisoprolol and was symptom free after its introduction (Figure 2). From our experience with exercise stress echocardiography conducted between 2002 and 2019 on 309 children [6], a significant orthostatic exercise-induced intraventricular gradient (Figure 1) of greater than 30 mmHg was observed in 101 (39%) of the 258 children evaluated due to distinct exercise-related symptoms, ECG abnormalities, or a positive stress ECG. These children were considered potential candidates for beta-blocker therapy [9]. The ST changes in the stress ECG of these children may be explained by subendocardial ischemia, which is associated with increased intraventricular pressure caused by exercise [11,12,13].

## Figures and Tables

**Figure 1:**
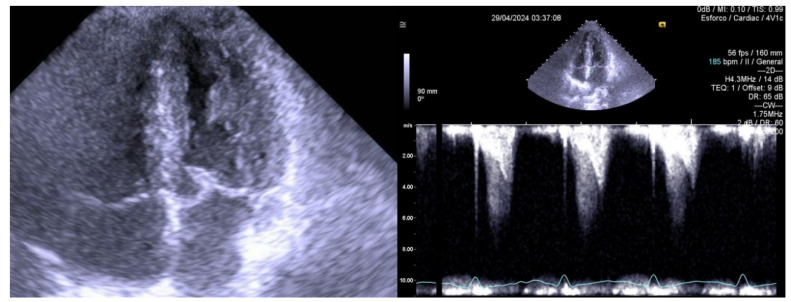
Peak exercise stress echocardiography off beta-blocker, with SAM (systolic anterior movement of the mitral valve (Left)), with intraventricular gradient (right), and with angina pectoris.

**Figure 2:**
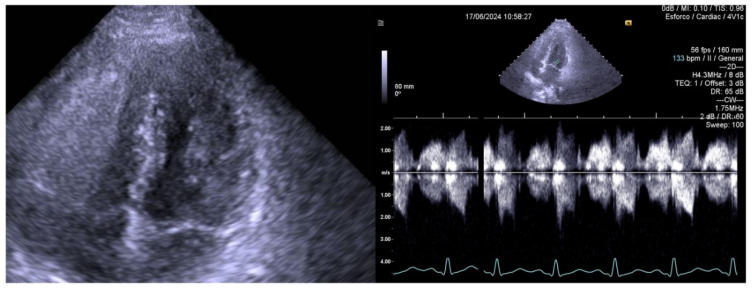
ECHO at the end of exercise stress echocardiography on bisoprolol 2.5 mg without SAM, without intraventricular gradient, and also without angina.
